# Genotype-Dependent Difference in 5-HT_2C_ Receptor-Induced Hypolocomotion: Comparison with 5-HT_2A_ Receptor Functional Activity

**DOI:** 10.1155/2015/846589

**Published:** 2015-08-26

**Authors:** Darya V. Bazovkina, Elena M. Kondaurova, Vladimir S. Naumenko, Evgeni Ponimaskin

**Affiliations:** ^1^Institute of Cytology and Genetics, Siberian Division of Russian Academy of Sciences, Novosibirsk 630090, Russia; ^2^Cellular Neurophysiology, Hannover Medical School, 30625 Hannover, Germany

## Abstract

In the present study behavioral effects of the 5-HT_2C_ serotonin receptor were investigated in different mouse strains. The 5-HT_2C_ receptor agonist MK-212 applied intraperitoneally induced significant dose-dependent reduction of distance traveled in the open field test in CBA/Lac mice. This effect was receptor-specific because it was inhibited by the 5-HT_2C_ receptor antagonist RS102221. To study the role of genotype in 5-HT_2C_ receptor-induced hypolocomotion, locomotor activity of seven inbred mouse strains was measured after MK-212 acute treatment. We found that the 5-HT_2C_ receptor stimulation by MK-212 decreased distance traveled in the open field test in CBA/Lac, C57Bl/6, C3H/He, and ICR mice, whereas it failed to affect locomotor activity in DBA/2J, Asn, and Balb/c mice. We also compared the interstrain differences in functional response to 5-HT_2C_ and 5-HT_2A_ receptors activation measured by the quantification of receptor-mediated head-twitches. These experiments revealed significant positive correlation between 5-HT2C and 5-HT2A receptors functional responses for all investigated mouse strains. Moreover, we found that 5-HT_2A_ receptor activation with DOI did not change locomotor activity in CBA/Lac mice. Taken together, our data indicate the implication of 5-HT_2C_ receptors in regulation of locomotor activity and suggest the shared mechanism for functional responses mediated by 5-HT_2C_ and 5-HT_2A_ receptors.

## 1. Introduction

The neurotransmitter serotonin (5-hydroxytryptamine, 5-HT) plays a crucial role in the mechanisms regulating a variety of brain functions. Serotonin effects in neurons are mediated by fourteen different receptor isoforms divided into seven groups (i.e., 5-HT_1_ to 5-HT_7_). With the exception of the 5-HT_3_ receptor, which is a transmitter-gated Na^+^/K^+^ channel, all other 5-HT receptors belong to a large family of G-protein coupled receptors (GPCRs). Among these receptors, 5-HT_2A_ and 5-HT_2C_ receptors are of particular interest because they are implicated in the regulation of multiple behavioral functions as well as in the etiology of affective disorders, including depression, anxiety, and obsessive-compulsive disorder [1–3].

The 5-HT_2C_ receptors are localized almost exclusively within the CNS with negligible expression in cardiac and vascular tissues. These receptors are widely distributed in the cortex, the limbic system, the basal ganglia, and especially the choroid plexus (tissue involved in production of cerebrospinal fluid) [4, 5]. From the functional point of view, the 5-HT_2C_ receptor is postulated to control feeding behavior [6], memory and learning [7], and sleep [8]. The 5-HT_2C_ agonists represent potential pharmacotherapeutics for the treatments of drug abuse and addiction [4, 9], while agomelatine, which is melatonergic agonist and 5-HT_2C_ antagonist, is effective for treatment of depression with relatively mild side effects [10]. In rodents, 5-HT_2C_ agonists can exert motor-suppression [11–13] and anxiogenic effect [14–16] in the elevated plus-maze and in the open field assays, respectively [17]. However, there is no data on the role of genotype for modulation of the 5-HT_2C_ receptor-induced behavioral responses.

Both 5-HT_2A_ and 5-HT_2C_ receptors show considerable homology in their primary and secondary structures as well as in their pharmacological profiles [18–21]. Although the 5-HT_2A_ and 5-HT_2C_ receptors activate the same cellular signal transduction pathways via phospholipase C (PLC) and phospholipase A2 [19], there are several lines of evidences showing that these closely related receptor subtypes could affect behavior in different ways. It has been shown that 5-HT_2A_ and 5-HT_2C_ receptors are involved in the neural control of male sexual motivation and arousal in rats by exerting reciprocal facilitative (5-HT_2A_) or suppressive (5-HT_2C_) influences [22]. Moreover, 5-HT_2A_ and 5-HT_2C_ antagonists affect the drinking behavior [23] and spatial reversal learning [24] in rats in the opposite ways. The distinct and in some cases opposite effects of 5-HT_2A_ and 5-HT_2C_ receptors have been also shown for various cocaine-mediated behavioral effects [25]. In addition, 5-HT_2A_ and 5-HT_2C_ receptors stimulation is differentially involved in the cortical dopamine efflux [26]. These data suggest the reciprocal function of 5-HT_2A_ and 5-HT_2C_ receptors in the brain.

The functional response of 5-HT_2A_ receptor can be determined by its activation with phenethylamine hallucinogens such as R(-)-2,5-dimethoxy-4-iodoamphetamine (DOI) which induces 5-HT_2A_ receptor-mediated head-twitch behavior in mice and rats [27–29]. However, it is still unknown whether a defined behavior can represent a specific read-out for the 5-HT_2C_ receptors activation, although a number of 5-HT_2C_ receptors-related behaviors are described. The goals of the present study were thus (i) to find behavioral effect of 5-HT_2C_ receptor activation which can be used as 5-HT_2C_ receptors specific read-out for functional response and (ii) to evaluate and compare the 5-HT_2A_ and 5-HT_2C_ receptors functional responses in different inbred mouse strains. For this purpose, we first examined the effects of 5-HT_2C_ receptors agonist in wide range of doses on behavior in the open field test. Secondly, we studied and compared the effect of 5-HT_2C_ receptor activation on locomotor activity with the effect of 5-HT_2A_ receptor activation on head-twitches number in seven inbred mouse strains.

## 2. Materials and Methods

### 2.1. Animals

Experiments were carried out on adult (10–12 weeks) male mice of CBA/Lac, C3H/He, C57Bl/6, ICR, DBA/2J, Asn, and Balb/c inbred strains. The mice were housed under standard laboratory conditions in a natural light-dark cycle (12 h light and 12 h dark) with free access to water and food. Two days prior to experiment the mice weighing about 25 g were isolated into individual cages to remove the group effect. All experimental procedures were in compliance with Guidelines for the Use of Animals in Neuroscience Research, 1992. All efforts were made to minimize the number of animals used and their sufferings. The study was carried out on the base of ICG SD RAS vivarium (RFMEFI61914X0005 and RFMEFI61914X0010).

### 2.2. Drugs

5-HT_2C_ receptor selective agonist MK-212 (2-chloro-6-(1-piperazinyl)pyrazine hydrochloride, 6-chloro-2-(1-piperazinyl)pyrazine hydrochloride, [30] Torcis Bioscience, UK) was dissolved in saline and administered intraperitoneally.

Selective 5-HT_2C_ receptor antagonist RS 102221 (8-[5-(2,4-dimethoxy-5-(4-trifluoromethylphenylsulphonamido)phenyl-5-oxopentyl)]-1,3,8-triazaspiro[4.5]decane-2,4-dione hydrochloride (Torcis Bioscience, UK)) was dissolved in the saline and administered intraperitoneally.

Preferential 5-HT_2A_ receptor agonist DOI ((±)-1-(2,5-dimethoxy-4-iodophenyl)-2-aminopropane hydrochloride, (±)-2,5-dimethoxy-4-iodoamphetamine hydrochloride (Sigma Aldrich, USA)) was dissolved in saline and administered intraperitoneally.

### 2.3. Open Field Test

The open field test was performed using round (40 cm in diameter) arena; the plastic walls were 25 cm high. The floor of the arena was made of mat and semitransparent plastic. The arena was placed on the mount at 40 cm above two halogen lamps of 12 W each. The light from the lamps diffused by the semitransparent floor was transmitted through the arena to the objective of digital camera (Panasonic) placed at 80 cm above the arena. A mouse was placed near the wall of the arena and tested during 5 minutes. The arena was carefully cleaned after each test. The video stream from the camera was analyzed frame-by-frame using the original EthoStudio software [31]. The total distance traveled (cm) and time spent in the center of arena were measured automatically for each mouse while amount of rearing and amount and time of grooming were measured by an observer blind to experimental design.

For estimation of 5-HT_2C_ receptors agonist effects on behavior in open field test the CBA/Lac mouse strain was used since high sensitivity to MK-212 was shown for these mice in separate experimental series: it was shown that MK-212 significantly reduced distance traveled in the open field. The efficiency of locomotor activity reduction was calculated as the ratio (%) of the difference between the mean distance traveled by mice treated with saline and the mean distance traveled by mice treated with MK-212 related to the mean distance traveled by saline group.

Consider *E* = (Ls − Lmk)*∗*100/Ls, where *E* represents the efficiency of locomotor activity reduction and Ls and Lmk represent the mean distance traveled by saline and MK-212 group, respectively.

To estimate the selectiveness of MK-212, this 5-HT_2C_ agonist was administered to mice of CBA strain pretreated with selective 5-HT_2C_ antagonist RS 102221 (2.0 mg/kg, i.p.) 30 min prior to the open field test and 10 min prior to MK-212 administration (2.0 mg/kg, i.p.). Optimal doses and time spans after drug administration were selected in separate experimental series where the wide range of doses (0.1, 1.0, 2.0, and 4.0 mg/kg) was utilized.

To study the possible influence of DOI on locomotor activity that could be mediated by the 5-HT_2C_ receptor, the effect of this drug on the behavior in the open field arena was estimated as well. DOI was administered intraperitoneally at doses 1.0 and 2.0 mg/kg 20 min prior to open field test. The distance traveled in the open field was measured according to above-described procedure.

### 2.4. Functional Activity of the 5-HT_2A_ Receptor

The 5-HT_2A_ receptor functional activity (sensitivity) was evaluated by the number of 5-HT_2A_ receptor-mediated head-twitches induced by intraperitoneal administration of DOI (1 mg/kg) as previously described [29]. The count of head-twitches was started 5 min after the drug administration and lasted for 20 min.

### 2.5. Statistical Analysis

The results were presented as mean (m) ± SEM or m + SEM and compared by means of one- or two-way ANOVA followed by post hoc Fisher's test. The interstrain correlation study was performed by Pearson correlation using strain means.

## 3. Results

### 3.1. Behavioral Effects of the 5-HT_2C_ Receptor in CBA/Lac Mouse Strain

**Figure 1:**
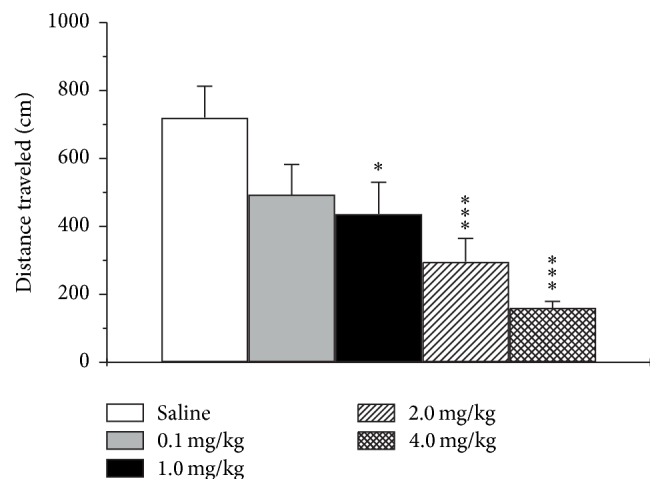
Effect of different doses of selective 5-HT_2C_ receptor agonist MK-212 on distance traveled in the open field test in CBA mice. The data are presented as the mean ± SEM (*n* = 8 per group; ^*∗*^ *p* < 0.05, ^*∗∗∗*^ *p* < 0.001 versus saline group).

**Figure 2:**
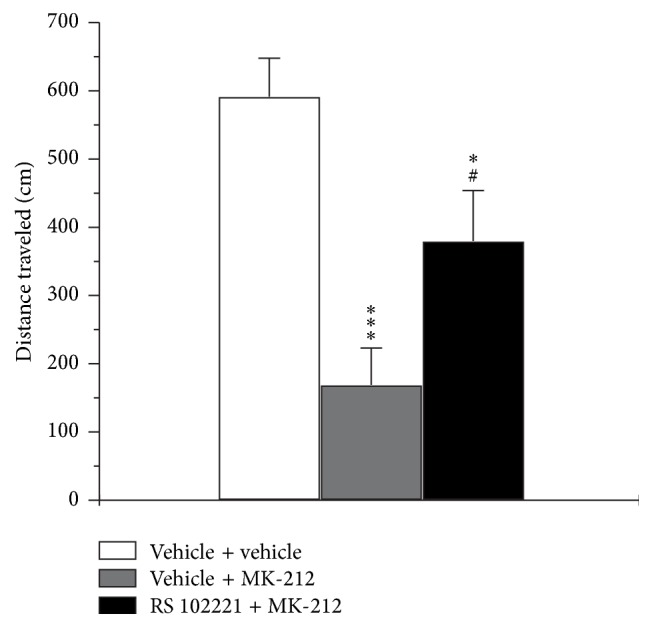
Effect of selective 5-HT_2C_ receptor antagonist RS 102221 on MK-212-induced hypolocomotion in the open field test in CBA mice. RS 102221 (2 mg/kg, i.p.) was administrated 10 min prior to MK-212 injection (2 mg/kg, i.p.) and 30 min prior to starting the behavioral test. The mice of control groups obtained double vehicle or vehicle plus MK-212 administration. The data are presented as mean ± SEM (*n* = 9 per group; ^*∗*^ *p* < 0.05, ^*∗∗∗*^ *p* < 0.001 compared with double vehicle group; ^#^ *p* < 0.05 compared with vehicle plus MK-212 group).

The open field test is widely used to evaluate the locomotor activity (total distance traveled in the open field arena) and anxiety-like behavior (time spent in the central part of the arena) in rodents [32, 33]. To analyze the role of 5-HT_2C_ receptor stimulation in modulation of locomotor activity, animals were subjected to the open field test after intraperitoneal injection of increasing concentrations of receptor agonist MK-212 (0.1, 1.0, 2.0, or 4.0 mg/kg). Administration of 5-HT_2C_ receptor agonist induced significant dose-dependent reduction of the distance traveled in the open field in mice of CBA/Lac strain (*F*_4,31_ = 6.2, *p* < 0.001; Figure 1). It is noteworthy that all doses, except for the lowest one (0.1 mg/kg), significantly decreased the path length, while highest MK-212 dose (4.0 mg/kg) produced almost threefold suppression of locomotor activity. For the time spent in the center, one-way ANOVA analysis did not show any effect of 5-HT_2C_ receptor agonist (*F*_4,31_ = 1.2, *p* > 0.05), but the post hoc analysis revealed significant decrease in this index in MK-212-treated (4.0 mg/kg) CBA mice compared to the saline group (*p* < 0.05; Table 1). MK-212-induced decrease in locomotor activity was 5-HT_2C_ receptor-specific, since the pretreatment of animal with i.p. injection of the highly selective 5-HT_2C_ receptor antagonist RS 102221 (2.0 mg/kg) significantly suppressed MK-212-induced reduction of distance traveled in the open field (*F*_2,22_ = 11.4; *p* < 0.001; Figure 2).

We also analyzed number of rearing events as a measure of exploratory behavior and anxiety [34]. Amount of rearing was significantly decreased after administration of 5-HT_2C_ receptor agonist (*F*_4,31_ = 6.2, *p* < 0.05; Table 1). Moreover, no rearing was observed after administration of the highest dose (4 mg/kg) of MK-212. In contrast, the 5-HT_2C_ receptor stimulation failed to affect grooming behavior, since neither amount (*F*_4,31_ = 1.3, *p* > 0.05) nor time of grooming in all utilized doses (*F*_4,31_ = 1.2, *p* > 0.05) manifested statistically different behavioral patterns (Table 1).

These combined results demonstrate that the total distance traveled in the open field arena represents the most convenient parameter for registration of the 5-HT_2C_ receptor functional response.

### 3.2. Role of Genotype in 5-HT_2C_ Receptor-Mediated Behavioral Effects

**Figure 3:**
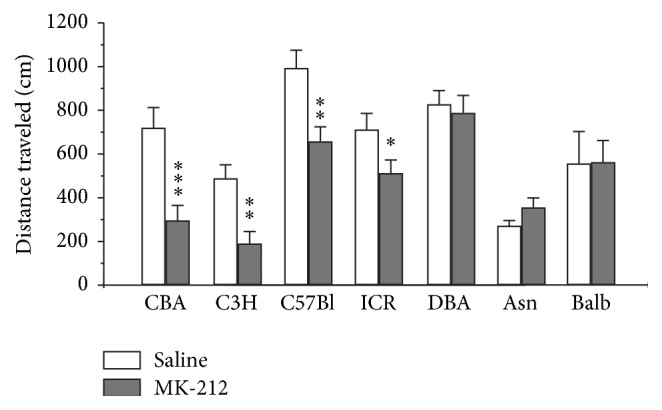
Effect of selective 5-HT_2C_ receptor agonist MK-212 (2 mg/kg, i.p.) on locomotor activity in the open field test in seven inbred mouse strains. The data are presented as mean ± SEM (*n* = 8 per group; ^*∗*^ *p* < 0.05, ^*∗∗*^ *p* < 0.01, and ^*∗∗∗*^ *p* < 0.001 compared with corresponding control).

**Figure 4:**
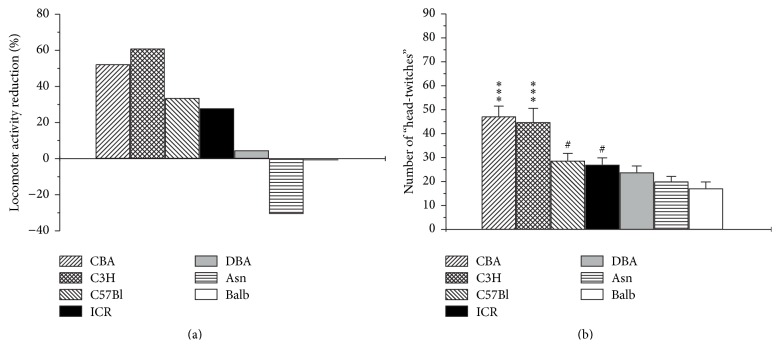
Functional activity of 5-HT_2A_ and 5-HT_2C_ receptors in seven inbred mouse strains. (a) The efficiency of locomotor activity reduction was calculated as the ratio (%) of the difference between the mean distance traveled by mice treated with saline and the mean distance traveled by mice treated with MK-212 related to the mean distance traveled by saline group. (b) Functional activity of 5-HT_2A_ receptor was estimated by number of 5-HT_2A_ receptor-mediated head-twitches induced by 5-HT_2A_ agonist DOI (1 mg/kg, i.p.). The data are presented as mean ± SEM (*n* = 8 per group; ^*∗∗∗*^ *p* < 0.001 compared with C57Bl/6, ICR, DBA/2J, Asn, and Balb/c strains; ^#^ *p* < 0.05 compared with Balb/c strain).

To study the role of genotype in behavioral effect mediated by the 5-HT_2C_ receptor, mice of seven inbred strains were tested in the open field test after acute treatment with MK-212 (i.p., 2.0 mg/kg; Figure 3). We have chosen the 2.0 mg/kg dose of MK-212 because it produced considerable, but not dramatic, decrease in distance traveled in the open field. In these experiments we obtained significant effect of the drug (*F*_1,93_ = 16.3, *p* < 0.001), genotype (*F*_6,93_ = 12.3, *p* < 0.001), and genotype × drug interaction (*F*_6,93_ = 3.0, *p* < 0.05) on distance traveled. It is however noteworthy that MK-212 decreased locomotor activity in the open field test only in CBA/Lac, C57Bl/6, C3H/He, and ICR strains with the most significant effect obtained in CBA/Lac and C3H/He mouse strains, in which MK-212 led to more than twofold reduction of locomotor activity (Figure 3). In contrast, the 5-HT_2C_ receptor stimulation did not alter the distance traveled in DBA/2J, Asn, and Balb/c strains (Figure 3). The decrease of distance traveled in the open field test was about 30 percent in C57Bl/6 and ICR animals treated with MK-212, when compared with corresponding saline groups (Figure 4(a)). Thus, 5-HT_2C_ receptor-mediated hypolocomotion is strongly dependent on genotype.

### 3.3. Comparison of 5-HT_2A_ and 5-HT_2C_ Behavioral Effects Along the Different Mouse Strains

To investigate a possible functional cross-reactivity between 5-HT_2A_ and 5-HT_2C_ receptors, the effect of 5-HT_2A_ receptor agonist DOI (1.0 and 2.0 mg/kg, i.p.) on locomotor activity was measured. These experiments revealed that both utilized doses of DOI were not effective in terms of reduction of distance traveled in the open field (*F*_2,17_ = 1.5, *p* > 0.05). The traveled path lengths were 986.3 ± 77.3, 1020.0 ± 57.6, and 828.9 ± 108.6 cm for 1.0 and 2.0 mg/kg DOI and for the control group, respectively.

In order to evaluate the role of genotype in modulation of the functional activity via the 5-HT_2A_ receptors and compare the latter with functional response of 5-HT_2C_ receptors, the number of 5-HT_2A_ receptor-mediated head-twitches induced by 5-HT_2A_ receptor agonist DOI (1.0 mg/kg, i.p.) was analyzed in different mouse strains. Treatment of animals with DOI produced head-twitches in all investigated mouse strains, but the intensity of this 5-HT_2A_ receptor-induced behavioral response was strain-dependent (*F*_6,63_ = 12.1, *p* < 0.001; Figure 4(b)). The most considerable effect was observed in CBA/Lac and C3H/He mice with 47.4 ± 4.2 and 45.0 ± 5.7 receptor-mediated head-twitches, respectively. The weakest effect of DOI was obtained in Balb/c mice (17.3 ± 2.6; Figure 4(b)).

**Figure 5:**
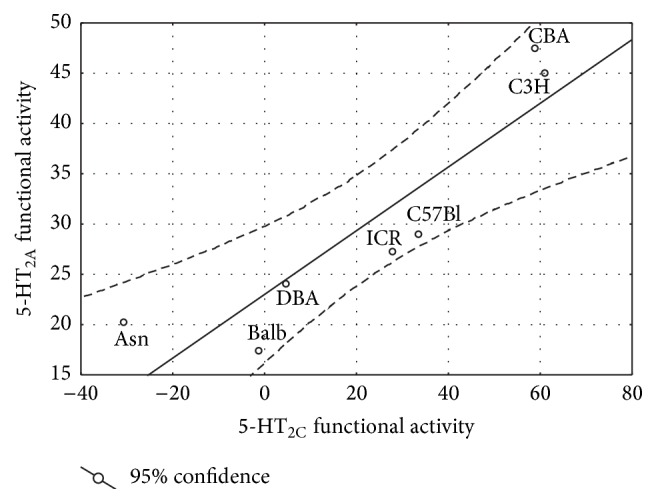
Correlation between 5-HT_2A_ and 5-HT_2C_ functional responses in seven inbred mouse strains. Correlation was estimated by the Pearson correlation using strain means. Correlation coefficient, *r* = 0.898.

Interestingly that comparison of interstrain differences in functional responses obtained after stimulation of the 5-HT_2A_ and 5-HT_2C_ receptors demonstrated the strong positive correlation (*r* = 0.898; *p* < 0.01; Figure 5) for all studied mouse strains.

## 4. Discussion

There is a body of evidences indicating that 5-HT_2C_ receptors activation produces locomotor-suppressant effect in rodents [12, 13, 30, 35–37], although some authors reported that only high doses of 5-HT_2C_ agonists reduced locomotion in the open field test [11]. In addition, agonists of the 5-HT_2C_ receptor such as mCPP and MK-212 induce the feeling of anxiety and panic in humans [38, 39] and anxiogenic-like behavior in animals [2, 11, 14, 15, 40]. Also the data obtained in transgenic and knockout mice indicate involvement of 5-HT_2C_ receptors in the regulation of both locomotor activity and anxiety. For example, transgenic mice overexpressing 5-HT_2C_ receptors in cerebral cortex and limbic areas showed decreased wheel-running behavior, reduced activity in the open field arena, and increased anxiety-like behavior in the elevated plus-maze [41]. Mutant mice lacking 5-HT_2C_ receptor are reported to exhibit an anxiolytic [42] and hyperactive [43] phenotype. Moreover, these animals were proposed as promising model of compulsive behavior [44].

In our study, selective 5-HT_2C_ agonist MK-212 induced significant dose-dependent reduction of distance traveled in the open field test in mice. This effect was 5-HT_2C_ receptor-specific because it was attenuated by the pretreatment of animals with the selective receptor antagonist RS 102221. It is noteworthy that only a high dose of MK-212 (4.0 mg/kg) reduced time spent in the center of open field arena that could be associated with anxiety-like behavior. Alternatively, this effect can also be explained by dramatic reduction of the total activity. This suggests that 5-HT_2C_ receptor-mediated reduction of locomotor activity represents a more sensitive 5-HT_2C_ receptors functional response than the increase in anxiety-like behavior.

Significant interstrain differences in the MK-212 influence on locomotor activity were found in seven inbred mouse strains. The most prominent effect was found in CBA/Lac strain suggesting a high 5-HT_2C_ receptor functional response in the brains of these mice. In contrast, MK-212 was not effective in DBA/2J, BALB/c, and Asn strains that suppose a low 5-HT_2C_ receptor functional response. Since MK-212 exhibits high efficacy and specificity for the 5-HT_2C_ receptors [45] the possible reason for the interstrain differences could be the different endophenotype related to genetic variance in serotonergic system. For example, it has been reported that C57Bl/6 mice exhibited a sensitized serotonin response to alcohol following repeated treatment, whereas sensitization was not observed in DBA2 animals [17]. Results of our study further confirm the important role of genetic background in behavioral response to 5-HT_2C_ receptor activation, although the precise mechanisms underlying the involvement of 5-HT_2C_ receptors in regulation of locomotor activity are not completely understood.

Considerable interstrain differences were also shown in number of head-twitches induced by 5-HT_2A_ receptors activation that implies the importance of genetic components in the functional state of 5-HT_2A_ receptors. Moreover, we found that the mouse strains demonstrating the high functional response of 5-HT_2C_ receptor also demonstrated high 5-HT_2A_ receptor-mediated functional activity and* vice versa*. The comparison of interstrain differences in 5-HT_2C_ receptor-mediated hypolocomotion with 5-HT_2A_ receptor-mediated head-twitches revealed the significant correlation between 5-HT_2C_ and 5-HT_2A_ receptor functional responses. These data suggest the shared mechanisms for the regulation of 5-HT_2C_ and 5-HT_2A_ receptors functional responses. At the same time, there are a lot of data on functional cross talk [46–49] as well as direct interaction between different serotonin receptors [50, 51]. For example, the functional cross talk between 5-HT_2A_ and 5-HT_1A_ receptor [47, 52] as well as between 5-HT_2A_ receptor and tryptophan hydroxylase-2 and 5-HT transporter [53] has been previously shown. Thus, the correlation between functional responses of 5-HT_2C_ and 5-HT_2A_ receptors might be mediated by the heterodimerization between these receptors, although this suggestion requires further investigations.

It has been shown that 5-HT_2A_ receptor agonist DOI at higher dose has an affinity to the 5-HT_2C_ receptor [27]. Here we demonstrated the specificity of 5-HT_2C_ receptors activation effect on locomotor activity, since 5-HT_2A_ receptor agonist DOI failed to affect locomotion behavior in the open field. These data are in agreement with the results described earlier [54]. On the other hand, it has been demonstrated that treatment of animal with DOI can increase distance traveled in the open field test [35, 37]. However, this increase was observed in the second half of 60 min observation interval after drug injection, while in present work we studied locomotor activity 20 min after DOI administration. Interestingly, only the high dose of DOI (10.0 mg/kg) produced reduction in locomotor activity suggesting that this response was mediated by 5-HT_2C_ receptors and not by the 5-HT_2A_ receptors. This interpretation is further supported by the finding that the DOI-induced hypolocomotion was blocked by pretreatment with selective 5-HT_2C/2B_ antagonist SER-082 [37]. Thus, these data suggest that 5-HT_2A_ and 5-HT_2C_ receptors could exert opposite effects on locomotor activity.

It is known that serotonin can modulate dopaminergic neurotransmission via 5-HT_2A_ and 5-HT_2C_ receptor subtypes [55–57]. The 5-HT_2A_ receptor agonists stimulate dopamine release [58, 59], while 5-HT_2C_ receptor agonists inhibit dopamine release from the mesoaccumbens and nigrostriatal projections upon agonist stimulation as well as under basal conditions [58, 60–62]. Taking into account the important role of brain dopamine system for the regulation of sensomotor integrity [63] and skeletal muscle contraction [64], the contrary effects of 5-HT_2A_ and 5-HT_2C_ receptor activation on locomotor activity could be attributed to the opposed regulatory influence on dopamine release regulated by these receptors. Moreover, since we found correlation between 5-HT_2C_ receptor-mediated hypolocomotion and 5-HT_2A_ receptor-mediated head-twitches, one could suggest that similar level of functional state of these receptor subtypes may be important for the serotonin-related regulation of dopamine release.

In conclusion, we demonstrated that acute administration of 5-HT_2C_ receptor agonist caused significant dose-dependent reduction of locomotor activity in mice. Thus, the intensity of 5-HT_2C_ receptor-mediated hypolocomotion could be used as a suitable read-out for the 5-HT_2C_ receptors functional state in the brain. In addition, comparison of 5-HT_2C_ receptor-induced reduction of locomotor activity in seven different inbred mouse strains was performed and the considerable difference in genetically defined 5-HT_2C_ receptor functional response was found. We also obtained the interstrain correlation of 5-HT_2C_ receptor-mediated hypolocomotion and 5-HT_2A_ receptor-mediated head-twitches number, suggesting the shared mechanisms for the regulation of 5-HT_2C_ and 5-HT_2A_ receptors functional responses.

## Figures and Tables

**Table 1:** Behavior of CBA mice treated with MK-212 and saline in the open field test.

	Time spent in center, %	Amount of rearing	Amount of grooming	Time of grooming
Saline	10.3 ± 4.3	4.8 ± 2.0	0.8 ± 0.3	4.1 ± 2.7
0.1	7.2 ± 3.5	2.5 ± 1.2	1.3 ± 0.4	2.1 ± 0.8
1.0	5.9 ± 1.9	1.0 ± 0.4^*∗*^	1.0 ± 0.4	5.1 ± 2.2
2.0	5.5 ± 2.6	1.2 ± 0.5^*∗*^	0.3 ± 0.2	1.1 ± 0.7
4.0	0.7 ± 0.4^*∗*^	0^*∗∗*^	0.6 ± 0.3	2.2 ± 1.2

Data are presented as the mean ± SEM (*n* = 8 per group). Time spent in center is presented as the percentage to total time of test. Statistically significant differences: ^*∗*^ *p* < 0.05, ^*∗∗*^ *p* < 0.01 versus saline group.
